# Clinical Utility of Prenatal cfDNA Screening for Sex Chromosome Aneuploidies: A Single Center Experience

**DOI:** 10.1002/mgg3.70211

**Published:** 2026-03-23

**Authors:** Ying Lin, Qun Lu, Yun Wu, Hang Li, Haoyang Feng, Chunyu Luo, Ping Hu, Dong Liang, Zhengfeng Xu

**Affiliations:** ^1^ Department of Prenatal Diagnosis Women's Hospital of Nanjing Medical University, Nanjing Women and Children's Healthcare Hospital Nanjing Jiangsu China; ^2^ Department of Ultrasound Women's Hospital of Nanjing Medical University, Nanjing Women and Children's Healthcare Hospital Nanjing Jiangsu China

**Keywords:** abnormal ultrasound, cfDNA screening, maternal sex chromosome aneuploidies, sex chromosome aneuploidies

## Abstract

**Introduction:**

To assess the clinical efficacy of prenatal cfDNA screening for detecting sex chromosome aneuploidies (SCAs).

**Material and Methods:**

We conducted a retrospective study at a single center including women with singleton pregnancies who underwent prenatal cfDNA screening between January 2020 and December 2024. Cases with high‐risk cfDNA screening results for SCAs were reviewed for confirmatory diagnostic results, prenatal ultrasound findings, and pregnancy outcomes. Positive predictive values (PPVs) were calculated for each SCA subtype, and associations between ultrasound findings and confirmed SCAs were analyzed.

**Results:**

A total of 252 pregnancies (252/58720, 0.43%) were identified as high‐risk cfDNA screening results for SCAs, including 224 fetal and 28 maternal cases. The fetal SCAs comprised 101 cases of 45,X, 37 of 47,XXX, 55 of 47,XXY, and 31 of 47,XYY. Among them, 173 underwent invasive diagnosis, confirming 90 true SCA cases with a PPV of 52.0%. For 45,X, there was a trend toward a higher detection rate in those with abnormal ultrasound findings compared to those with normal ultrasound results (4/20 vs. 8/127, *p* = 0.060), though it did not reach statistical significance; no significant differences were observed for sex chromosome trisomies. Of the 16 maternal SCA cases that underwent invasive testing, all fetuses had normal karyotypes.

**Conclusions:**

Our findings demonstrated prenatal cfDNA screening as a unique approach to achieve effective detection of fetal SCAs, particularly those that otherwise would be overlooked by routine prenatal ultrasound screening.

## Graphical abstract

CfDNA screening effectively detected SCAs often undetected by routine prenatal ultrasound. Maternal SCAs showed rare fetal transmission.

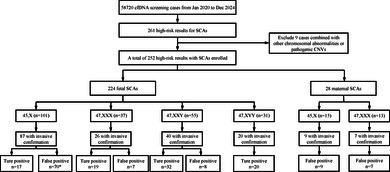

**Abbreviations**
CfDNA: cell‐free DNA
PPV: positive predictive value
SCAs: sex chromosome aneuploidies
SCTs: sex chromosome trisomies

## 1 Introduction

Sex chromosome aneuploidies (SCAs), including monosomy X (45,X), and sex chromosome trisomies (SCTs) (47,XXX; 47,XXY; 47,XYY), are among the most common chromosomal abnormalities, affecting approximately 1 in 400 live births (Reimers et al. 2023). There are no pathognomonic features of SCAs, and there is great variability in penetrance and expressivity, affecting growth, gonadal function, and neurodevelopment (Reimers et al. 2023; Green et al. 2019; Urbanus, Swaab, et al. 2020). Early recognition, followed by timely intervention and proper postnatal management, has the potential to improve the quality of life for affected individuals (Pieters et al. 2011; Grynberg et al. 2016; Flannigan et al. 2018; Wigby et al. 2016; Urbanus, van Rijn, and Swaab 2020).

Prenatal cell‐free DNA (cfDNA) analysis has transformed prenatal screening for common trisomies and is increasingly used to screen for other abnormalities, including SCAs. Studies reported that the sensitivity and specificity of prenatal cfDNA screening for SCAs were 94.1% and 99.5% (Bussolaro et al. 2023). Professional guidelines of the American College of Medical Genetics and Genomics (ACMG, 2023) and the Society for Maternal‐Fetal Medicine (SMFM, 2025) acknowledge that cfDNA screening provides a unique opportunity to identify fetal SCAs (Dungan et al. 2023; Society for Maternal‐Fetal Medicine et al. 2025). Although widespread clinical adoption has markedly increased the rate of prenatal SCA detection in recent years (Samango‐Sprouse et al. 2023), yet several aspects remain insufficiently characterized, including cfDNA screening performance for SCA subtypes, the impact of maternal SCAs on fetal outcomes, and its added value compared with prenatal ultrasound.

In this study, we report on the experience of a single center in which SCAs were routinely included in prenatal cfDNA screening. Leveraging systematic follow‐up and detailed clinical data, we assessed test performance for SCA subtypes, examined how incidentally detected maternal SCAs may confound interpretation, and explored the diagnostic contribution of prenatal ultrasound. These analyses aim to clarify the clinical value of cfDNA screening for SCAs in routine prenatal screening practice.

## 2 Materials and Methods

### 2.1 Study Design and Participants

In this single center study, we reviewed all prenatal cfDNA screening cases conducted between January 2020 and December 2024 in the Center of Prenatal Diagnosis at Women's Hospital of Nanjing Medical University. In our center, cfDNA screening was offered as a routine prenatal aneuploidy screening strategy to pregnant women between 12^+0^ and 26^+6^ weeks of gestation, excluding those with a history of organ transplantation, fetal structural abnormalities, vanished twin, pregnancy with malignant tumors, or multiple gestations. We collected all pregnancies with high‐risk cfDNA screening results for SCAs. Cases involving other chromosomal aneuploidies or pathogenic copy number variations (CNVs) were excluded. Informed consent was obtained from all participants.

### 2.2 Prenatal cfDNA Sequencing and Genetic Confirmation

Cell‐free DNA extraction, library preparation, and sequencing were performed as previously described (Liang et al. 2018). Sequencing was carried out on the BGISEQ‐500 and MGISEQ‐2000 platforms using single‐end 35‐bp reads, which were aligned to the human reference genome (hg19, NCBI build 37). Samples were required to meet quality control thresholds of unique reads ≥ 3.5 Mb, GC content (referring to the proportion of G and C bases among the total bases in the sequencing reads) between 38% and 42%, and a fetal fraction ≥ 3.5%. CfDNA screening high‐risk results for SCAs included 45,X, 47,XXX, 47,XXY, and 47,XYY. SCAs were assessed by quantifying dosage deviations of the sex chromosomes relative to autosomal references to calculate the relative fetal chromosomal concentrations of X and Y (*F*_*X*_ and *F*_*Y*_), consistent with previously reported approaches (Yao et al. 2014, 2012). Interpretation was based on the joint distribution of *F*_*X*_ and *F*_*Y*_: normal female fetuses (46,XX) were characterized by *F*_*X*_≈0 and *F*_*Y*_≈0, whereas normal male fetuses (46,XY) exhibited *F*_*X*_ > 0 and *F*_*Y*_ > 0. Distinct dosage patterns were observed for different SCAs: 45,X was characterized by *F*_*X*_ > 0 and *F*_*Y*_≈0; 47,XXX by *F*_*X*_ < 0 and *F*_*Y*_≈0; 47,XXY by *F*_*X*_≈0 and *F*_*Y*_ > 0; and 47,XYY by *F*_*X*_ > 0 with *F*_*Y*_ markedly exceeding *F*_*X*_, with *F*_*Y*_ approximately twice those of *F*_*X*_. In addition, extreme deviations of *F*_*X*_ (e.g., > 40% or < −40%) suggested potential interference from maternal SCA (such as 45,X or 47,XXX).

Pregnant women identified as high‐risk cfDNA screening for SCA were referred for genetic counseling, and confirmatory testing was recommended via amniocentesis, followed by karyotyping or chromosomal microarray analysis (CMA).

### 2.3 Clinical Information Collected

During pretest counseling for prenatal cfDNA screening, we collected first‐trimester ultrasound findings, particularly nuchal translucency (NT) measurements, whenever available. Following cfDNA screening, additional information was obtained through the outpatient medical record system or telephone follow‐up, including second‐ and third‐trimester ultrasound results and amniotic fluid genetic testing results. For pregnancy outcome follow‐up, we relied on birth registration records and detailed physical examination reports a month after delivery to assess the health status of the newborns.

### 2.4 Statistical Analysis

Continuous variables, such as maternal age, gestational age, and body mass index, were reported as mean ± standard deviation. Fisher's exact test was used to assess between‐group differences in the distribution of findings. A *p*‐value < 0.05 was considered statistically significant. All statistical analyses were performed using the R software (version 4.1.0).

## 3 Results

### 3.1 Characteristics of Study Population

**FIGURE 1:**
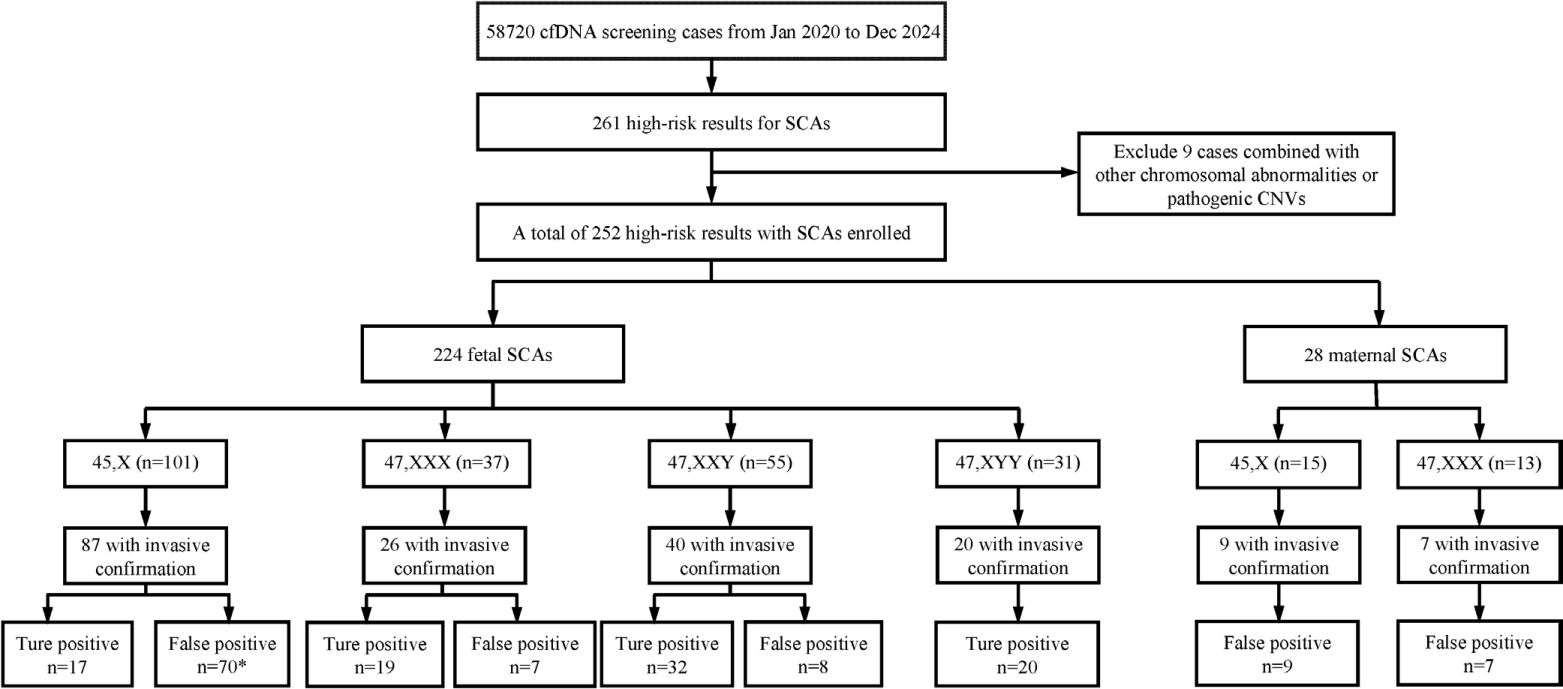
Study design and validated results of high‐risk SCA results. *Two with 47,XXX. SCAs, sex chromosome aneuploidies; CNVs, copy number variations.

A total of 58,720 singleton pregnancies underwent prenatal cfDNA screening from January 2020 to December 2024 at our center. After excluding nine cases with other chromosomal aneuploidies or pathogenic CNVs, 252 pregnancies (0.43%) were identified as high risk for SCAs. Among the 252 cases, 224 were classified as fetal SCAs, including 101 cases of 45,X; 37 of 47,XXX; 55 of 47,XXY and 31 of 47,XYY. The remaining 28 cases were maternal SCAs, comprising 15 cases of 45,X and 13 cases of 47,XXX (Figure 1). Baseline maternal characteristics are presented in Table 1, the mean maternal age was 30.9 years (range 20–46), and the mean gestational age at testing was 16^+5^ weeks (range 12^+0^–26^+6^).

**TABLE 1:** Characteristics of the study population by prenatal cfDNA screening risk classification.

Sample characteristics	High‐risk for fetal SCAs (*n* = 224)	High‐risk for maternal SCAs (*n* = 28)
Mean gestational age (SD), week	16.64 (2.46)	17.20 (2.47)
Mean maternal age (SD), year	31.10 (4.01)	29.25 (4.08)
Mean BMI (SD), g/m2	22.40 (3.06)	23.84 (3.99)
Number of pregnancies (SD)	1.85 (1.07)	2.16 (1.74)
Fetal fraction (SD), %	12.71 (7.55)	10.70 (6.91)

Abbreviations: BMI, body mass index; SCAs, sex chromosome aneuploidies.

### 3.2 Genetic Confirmation of SCAs

Among the 224 high‐risk fetal SCAs, 173 cases chose invasive prenatal diagnosis. A total of 90 cases were confirmed as true SCAs, and 83 cases were found to have normal karyotypes, yielding an overall positive predictive value (PPV) of 52.0% (90/173). Additionally, there were two cases—originally flagged as high‐risk cfDNA screening results for 45,X—which were found to carry 47,XXX. Predictive accuracy varied substantially across subtypes, with 45,X showing the lowest PPV at 19.5% (17/87), while SCTs demonstrated much higher values, ranging from 73.1% (19/26) for 47,XXX to 100% (20/20) for 47,XYY (Figure 1). These findings indicate that the reliability of cfDNA screening‐detected high‐risk SCAs depends heavily on the subtype involved.

In addition to fetal SCAs, 28 cases were identified as maternal SCAs. Of these, 16 underwent invasive prenatal diagnosis, and all fetal karyotypes were normal (Figure 1). These findings revealed that maternal SCAs detected by prenatal cfDNA screening were not associated with fetal SCAs in our series.

### 3.3 Prenatal Ultrasound Findings

Among the confirmed cases, ultrasound findings including first‐trimester NT measurement or second‐ to third‐trimester ultrasound scanning were available for 85.0% (147/173) cases. Among true‐positive 45,X cases, 4 of 12 (33.3%) showed abnormal findings, including one case with increased NT (> 3.0 mm), one with a ventricular septal defect, and two with shortened long bones (< 5th percentile for gestational age). In comparison, 10 of 55 (18.2%) false‐positive 45,X cases presented with ultrasound anomalies (*p* = 0.257). For 47,XXX, 47,XXY, and 47,XYY, the proportions of abnormal findings among true positives were 10.5%, 7.4%, and 10.5%, respectively, with all false positives showing normal ultrasound results (all *p* = 0.999) (Tables S1 and S2). Overall, the presence of ultrasound anomalies did not significantly differ between true‐ and false‐positive cases for any SCA subtype and full individual‐level ultrasound results are detailed in Table S3.

### 3.4 Ultrasound‐Based Detection Estimates Without Prenatal cfDNA Screening Integration Among cfDNA Screening High‐Risk Pregnancies

**FIGURE 2:**
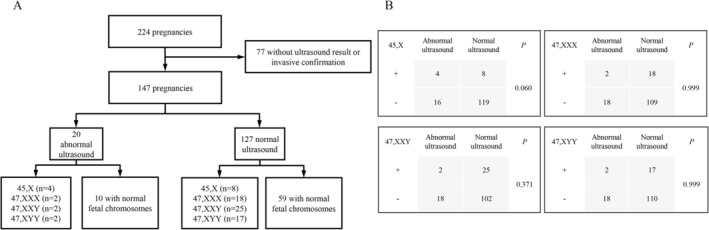
Detection of SCAs based on fetal ultrasound findings. (A) Flowchart of ultrasound findings and confirmed fetal SCAs. (B) Comparison of the detection rate of SCAs between normal and abnormal ultrasound cases. SCAs, sex chromosome aneuploidies.

To explore a counterfactual clinical scenario in which prenatal cfDNA screening was unavailable, we analyzed ultrasound findings among pregnancies that were identified as high risk for fetal SCAs by cfDNA screening, simulating prenatal diagnostic decision‐making guided solely by ultrasound abnormalities. We analyzed all 224 pregnancies flagged as high‐risk fetal SCAs by cfDNA screening. After excluding 77 cases without available ultrasound results or confirmatory invasive testing, 147 pregnancies remained for analysis. Among these, 20 fetuses presented with abnormal ultrasound findings. If invasive procedures had been performed based only on these abnormal ultrasound findings, 10 SCAs would have been detected: four cases of 45,X; two of 47,XXX; two of 47,XXY and two of 47,XYY. However, this approach would have missed 68 SCAs among the remaining 127 fetuses with normal ultrasound findings, including eight cases of 45,X; 18 of 47,XXX; 25 of 47,XXY and 17 of 47,XYY (Figure 2A). The detection rate for 45,X was numerically higher among fetuses with abnormal ultrasound compared to those with normal findings (4/20 vs. 8/127, *p* = 0.060), although the difference was not statistically significant. In contrast, there was no appreciable difference in detection rates for 47,XXX; 47,XXY or 47,XYY between the two groups (Figure 2B), indicating that ultrasound alone has poor sensitivity for detecting SCTs. These findings reveal the clinical value of prenatal cfDNA screening in identifying SCAs, particularly for subtypes with subtle or absent prenatal phenotypes.

### 3.5 Pregnancy Outcomes

Pregnancy decisions varied substantially across SCA subtypes. Among confirmed cases, termination was common for 45,X and 47,XXY, with 76.5% (13/17) and 84.4% (27/32) of pregnancies electively terminated, respectively. In contrast, the majority of 47,XXX (15/19) and 47,XYY (12/20) cases resulted in live births. In false‐positive cases across all subtypes, the live birth rate was 91.6% (76/83), and only one termination was reported for nonmedical reasons. These patterns likely reflect differing parental perceptions and counseling outcomes related to each SCA subtype. Full details of pregnancy outcomes are provided in Table S3.

## 4 Discussion

This large‐scale retrospective study provides a real‐world evaluation of prenatal cfDNA screening for SCAs in 58,720 singleton pregnancies. Our findings demonstrate that cfDNA screening reliably detects fetal SCAs, including those with subtle or absent ultrasound findings, which are often missed by routine prenatal ultrasound, highlighting its added value in prenatal screening. The overall PPV for fetal SCAs was 50.9%, which varied markedly across subtypes from 19.5% for 45,X to 100% for 47,XYY, reflecting the subtype‐dependent performance of cfDNA screening. Maternal SCAs were detected in approximately 0.05% (16/58720) pregnancies, with rare (0 out of 16) transmission to the fetus. These results provide valuable evidence on the clinical utility of cfDNA screening for SCAs, supporting its role in guiding prenatal counseling and risk assessment across different SCA subtypes.

Compared with ultrasound‐based screening, prenatal cfDNA screening demonstrated higher sensitivity in detecting SCAs, particularly SCTs, which often lack specific ultrasound findings (Luo et al. 2024). In our cohort, most confirmed SCTs would have been missed if invasive testing had been guided solely by abnormal ultrasound findings, highlighting the limited detection yield of ultrasound for these conditions. Similarly, abnormal ultrasound findings for 45,X were also limited in our cohort, partly due to the presence of mosaicism in most 45,X cases (16/17) and the fact that cfDNA sampling occurred primarily in the second trimester. Such circumstances reduce the likelihood of observing classic early ultrasound findings like increased NT or cystic hygroma (Reimers et al. 2023; Hook and Warburton 1983; Sybert and McCauley 2004; Gruchy et al. 2014; Bedei et al. 2023). Taken together, these observations suggest that many SCAs present with subtle or absent ultrasound findings, which may limit the effect of ultrasound screening when used alone. CfDNA screening provides a complement to routine imaging by enhancing detection rates and informing clinical management. Aligned with the 2025 SMFM guideline, a positive cfDNA screening result of SCAs warrants the offer of confirmatory diagnostic testing, regardless of the ultrasound findings (Society for Maternal‐Fetal Medicine et al. 2025).

Consistent with previous studies, reproductive decisions varied substantially across SCA subtypes in our cohort, with higher termination rates for 45,X and 47,XXY than for 47,XXX and 47,XYY (Mezei et al. 2004; Lu et al. 2021). These subtype‐specific differences likely reflect parental perceptions shaped by phenotype severity and genetic counseling after a positive result. While 45,X and 47,XXY are associated with more defined clinical implications, 47,XXX and 47,XYY typically present with milder and more variable manifestations (Mezei et al. 2004; Jeon et al. 2012). These findings underscore the importance of comprehensive posttest counseling that considers SCA subtype and expected clinical outcomes to support informed parental decision‐making.

Among the 28 pregnancies as maternal SCAs, over half underwent invasive prenatal diagnosis, and none of the fetuses were found to carry SCAs, supporting a generally reassuring fetal prognosis. This finding aligns with previous reports showing that most maternal SCAs have chromosomally normal children, although rare recurrence has been reported due to gonadal mosaicism or structural rearrangements (Reimers et al. 2023) (Hadnott et al. 2011) (Bernard et al. 2016). Although guidelines recommend prenatal diagnostic for fetal in cases of maternal‐origin SCAs (Dungan et al. 2023), our results suggested that the risk of fetal involvement is low, which could add information for proper posttest genetic counseling. Nonetheless, women with maternal SCAs may warrant closer antenatal monitoring due to potential obstetric complications (Hadnott et al. 2011).

Our study also has several limitations. First, a proportion of prenatal cfDNA screening high‐risk cases did not undergo confirmatory diagnostic testing, which may have introduced verification bias. Second, long‐term developmental outcomes were not systematically assessed, restricting the evaluation of the broader clinical value of prenatal cfDNA screening for SCAs. Further studies with standardized diagnostic confirmation and postnatal follow‐up are needed to validate these findings.

## 5 Conclusion

In conclusion, our findings highlight the added clinical value of cfDNA screening in prenatal care, as it enables the detection of SCAs that often elude ultrasound screening. Additionally, maternal SCAs detected by prenatal cfDNA screening are seldom transmitted to the fetus, providing information for proper clinical counseling and risk assessment.

## Author Contributions

Prepared the manuscript: Ying Lin, Qun Lu; design of the article: Dong Liang, Zhengfeng Xu; collected samples: Hang Li, Chunyu Luo, Ping Hu; analyzed or interpreted the data: Ying Lin, Qun Lu, Dong Liang, Yun Wu; collected clinical information: Yun Wu, Zhengfeng Xu; DNA extraction, library construction, and sequencing: Hang Li, Haoyang Feng.

## Funding

This work was supported by the National Key R&D Program of China (grant number: 2022YFC2703400); the Jiangsu Province Capability Improvement Project through Science, Technology and Education Jiangsu Provincial Medical Key Discipline (grant number: ZDXK202211); Research Project of Jiangsu Province Association of Maternal and Child Health (grant number: FYX202312).

## Ethics Statement

This study was ethically approved by the Medical Ethics Committee of Women's Hospital of Nanjing Medical University (Reference number NFLZ 2017–120; Date of approval 12.05.2017).

## Conflicts of Interest

The authors declare no conflicts of interest.

## Supporting information


**Table S1:** Comparison of ultrasound findings between true‐positive and false‐positive in high‐risk results of fetal SCAs.

**Table S2:** Summary of ultrasound findings in fetuses with confirmed SCAs.

**Table S3:** The confirmatory diagnostic results, ultrasound screening results and pregnancy outcomes in 252 high‐risk SCA cases.

## Data Availability

The data that support the findings of this study are available on request from the corresponding author. The data are not publicly available due to privacy or ethical restrictions.
