# Supplementary material for: Clinical Utility of Prenatal cfDNA Screening for Sex Chromosome Aneuploidies: A Single Center Experience
Source: Mol Genet Genomic Med. 2026 Mar 23;14(3):e70211. doi: 10.1002/mgg3.70211 (PMC13097572; doi:10.1002/mgg3.70211)
Supplement: Supplementary file 1 — Table S1: Comparison of ultrasound findings between true‐positive and false‐positive in high‐risk results of fetal SCAs. [file MGG3-14-e70211-s002.docx]

| SCA | Invasive confirmation | N | Ultrasound findings | | *P* value |
| --- | --- | --- | --- | --- | --- |
|  |  |  | Normal ultrasound | Abnormal ultrasound |  |
| 45,X | True positive | 17 | 8 | 4 | 0.257 |
|  | False positive | 67 | 45 | 10 |  |
| 47,XXX | True positive | 19 | 17 | 2 | 0.999 |
|  | False positive | 7 | 7 | 0 |  |
| 47,XXY | True positive | 32 | 25 | 2 | 0.999 |
|  | False positive | 8 | 6 | 0 |  |
| 47,XYY | True positive | 20 | 17 | 2 | / |
|  | False positive | 0 | 0 | 0 |  |

Table S1 Comparison of ultrasound findings between true positive and false positive in

high-risk results of fetal SCAs.

SCA, sex chromosome aneuploidies.
