# Supplementary material for: Clinical Utility of Prenatal cfDNA Screening for Sex Chromosome Aneuploidies: A Single Center Experience
Source: Mol Genet Genomic Med. 2026 Mar 23;14(3):e70211. doi: 10.1002/mgg3.70211 (PMC13097572; doi:10.1002/mgg3.70211)
Supplement: Supplementary file 2 — Table S2: Summary of ultrasound findings in fetuses with confirmed SCAs. [file MGG3-14-e70211-s003.docx]

Table S2. Summary of ultrasound findings in fetuses with confirmed SCAs.

| SCA subtype | Confirmed SCAs | With available ultrasound results | With abnormal ultrasound findings (%) | Abnormal ultrasound finding |
| --- | --- | --- | --- | --- |
| 45,X | 17 | 12 | 4 (33.3%） | 1 increased nuchal translucency  2 Shortened long bones  1 VSD |
| 47,XXX | 20 | 19 | 2 (10.5%） | 1 Unilateral choroid plexus cyst  1 VSD |
| 47,XXY | 32 | 27 | 2 (7.4%） | 1 VSD, Talipes equinovarus  1 FGR |
| 47,XYY | 20 | 19 | 2 (10.5%) | 1 VSD  1 Choroid plexus cyst |

SCAs, sex chromosome aneuploidies; VSD, ventricular septal defect; FGR, fetal growth restriction.
