# Supplementary material for: Clinical Utility of Prenatal cfDNA Screening for Sex Chromosome Aneuploidies: A Single Center Experience
Source: Mol Genet Genomic Med. 2026 Mar 23;14(3):e70211. doi: 10.1002/mgg3.70211 (PMC13097572; doi:10.1002/mgg3.70211)
Supplement: Supplementary file 3 — Table S3: The confirmatory diagnostic results, ultrasound screening results and pregnancy outcomes in 252 high‐risk SCA cases. [file MGG3-14-e70211-s001.docx]

Table S3. The confirmatory diagnostic results, ultrasound screening results and pregnancy outcomes in 252 high-risk SCA cases.

| Case | Age | Gestational age of cfDNA screening | cfDNA screening results | cfDNA screening classification | Confirmatory diagnostic results | Ultrasound results | Pregnancy outcome |
| --- | --- | --- | --- | --- | --- | --- | --- |
| 1 | 28 | 17+1 | 47,XYY | True positive | arr[hg19](1-22,Y)×2,(X)×1 | Normal | Live birth |
| 2 | 33 | 20+1 | 47,XYY | True positive | arr[hg19](1-22,Y)×2,(X)×1 | Normal | Live birth |
| 3 | 30 | 17+1 | 47,XYY | True positive | 47,XYY | Normal | Live birth |
| 4 | 33 | 17+2 | 47,XYY | True positive | 47,XYY | Normal | TOP |
| 5 | 34 | 12+3 | 47,XYY | True positive | 47,XYY | Normal | Live birth |
| 6 | 38 | 15+6 | 47,XYY | True positive | arr[hg19](1-22,Y)×2,(X)×1 | NA | Live birth |
| 7 | 28 | 18+3 | 47,XYY | True positive | arr[hg19](1-22,Y)×2,(X)×1 | Normal | Live birth |
| 8 | 26 | 16+0 | 47,XYY | - | NA | Normal | Live birth |
| 9 | 33 | 16+4 | 47,XYY | True positive | arr[hg19](1-22,Y)×2,(X)×1 | Normal | Live birth |
| 10 | 31 | 18+3 | 47,XYY | - | NA | Normal | Live birth |
| 11 | 27 | 18+5 | 47,XYY | - | NA | NA | Live birth |
| 12 | 28 | 15+2 | 47,XYY | - | NA | NA | NA |
| 13 | 30 | 13+2 | 47,XYY | True positive | 47,XYY | Normal | Live birth |
| 14 | 34 | 16+6 | 47,XYY | - | NA | Normal | Live birth |
| 15 | 37 | 16+5 | 47,XYY | True positive | 47,XYY | Normal | NA |
| 16 | 27 | 16+1 | 47,XYY | True positive | 47,XYY | Normal | Live birth |
| 17 | 34 | 12+4 | 47,XYY | - | NA | Normal | Live birth |
| 18 | 29 | 15+5 | 47,XYY | True positive | 47,XYY | Normal | TOP |
| 19 | 33 | 16+0 | 47,XYY | True positive | 47,XYY | Normal | Live birth |
| 20 | 28 | 17+1 | 47,XYY | - | NA | Normal | Live birth |
| 21 | 41 | 16+4 | 47,XYY | True positive | 47,XYY | VSD 2.1mm | Live birth |
| 22 | 33 | 14+0 | 47,XYY | - | NA | Normal | Live birth |
| 23 | 31 | 21+3 | 47,XYY | - | NA | Normal | Live birth |
| 24 | 33 | 14+0 | 47,XYY | True positive | 47,XYY | Normal | Live birth |
| 25 | 35 | 16+5 | 47,XYY | - | NA | Normal | Live birth |
| 26 | 31 | 16+5 | 47,XYY | True positive | 47,XYY | Normal | Miscarriage |
| 27 | 26 | 12+2 | 47,XYY | True positive | 47,XYY | Choroid plexus cyst | TOP |
| 28 | 29 | 12+3 | 47,XYY | - | NA | Normal | Live birth |
| 29 | 27 | 13+2 | 47,XYY | True positive | 47,XYY | Normal | TOP |
| 30 | 30 | 17+5 | 47,XYY | True positive | 47,XYY | Normal | NA |
| 31 | 38 | 14+4 | 47,XYY | True positive | 47,XYY | Normal | TOP |
| 32 | 38 | 17+0 | 47,XXY | True positive | 47,XYY/48,XXYY | VSD 2.2mm, Talipes equinovarus | TOP |
| 33 | 34 | 16+0 | 47,XXY | True positive | 47,XXY | Normal | TOP |
| 34 | 31 | 18+4 | 47,XXY | False positive | 46,XY | Normal | Live birth |
| 35 | 31 | 16+3 | 47,XXY | - | NA | Normal | Live birth |
| 36 | 36 | 16+0 | 47,XXY | True positive | arr[hg19](1-22,X)×2,(Y)×1 | FGR | TOP |
| 37 | 35 | 17+3 | 47,XXY | True positive | 47,XXY | Normal | TOP |
| 38 | 28 | 16+0 | 47,XXY | True positive | arr[hg19](1-22,X)×2,(Y)×1 | Normal | TOP |
| 39 | 31 | 17+0 | 47,XXY | True positive | 47,XXY | Normal | TOP |
| 40 | 35 | 16+5 | 47,XXY | True positive | arr[hg19](1-22,X)×2,(Y)×1 | Normal | TOP |
| 41 | 38 | 19+0 | 47,XXY | False positive | 46,XY | NA | Live birth |
| 42 | 34 | 17+3 | 47,XXY | - | NA | NA | TOP |
| 43 | 25 | 18+5 | 47,XXY | True positive | 47,XXY | NA | TOP |
| 44 | 27 | 16+4 | 47,XXY | False positive | 46,XY | NA | TOP |
| 45 | 28 | 16+1 | 47,XXY | True positive | arr[hg19](1-22,X)×2,(Y)×1 | Normal | TOP |
| 46 | 33 | 16+0 | 47,XXY | - | NA | VSD 2mm | NA |
| 47 | 27 | 12+2 | 47,XXY | - | NA | Normal | Live birth |
| 48 | 33 | 16+4 | 47,XXY | False positive | 46,XY | Normal | Live birth |
| 49 | 31 | 16+3 | 47,XXY | True positive | arr[hg19](1-22,X)×2,(Y)×1 | NA | TOP |
| 50 | 24 | 17+5 | 47,XXY | - | NA | Normal | NA |
| 51 | 40 | 16+5 | 47,XXY | True positive | 47,XXY | Normal | TOP |
| 52 | 34 | 12+3 | 47,XXY | True positive | 47,XXY | Normal | TOP |
| 53 | 27 | 16+2 | 47,XXY | - | NA | Normal | Live birth |
| 54 | 29 | 16+0 | 47,XXY | True positive | 47,XXY | Normal | TOP |
| 55 | 26 | 16+4 | 47,XXY | False positive | 46,XX | Normal | Live birth |
| 56 | 35 | 16+2 | 47,XXY | True positive | 47,XXY | Normal | TOP |
| 57 | 31 | 16+2 | 47,XXY | False positive | 46,XY | Normal | Live birth |
| 58 | 24 | 17+3 | 47,XXY | True positive | arr[hg19](1-22,X)×2,(Y)×1 | Normal | Live birth |
| 59 | 39 | 16+6 | 47,XXY | True positive | 47,XXY | Normal | Live birth |
| 60 | 29 | 19+3 | 47,XXY | False positive | 46,XY | Normal | Live birth |
| 61 | 33 | 16+0 | 47,XXY | True positive | 47,XXY | Normal | TOP |
| 62 | 31 | 16+4 | 47,XXY | True positive | 47,XXY | Normal | TOP |
| 63 | 36 | 16+2 | 47,XXY | - | NA | NA | NA |
| 64 | 34 | 15+5 | 47,XXY | True positive | 47,XXY | NA | TOP |
| 65 | 25 | 24+1 | 47,XXY | True positive | 47,XXY | Normal | TOP |
| 66 | 32 | 15+3 | 47,XXY | False positive | 46,XY | Normal | Live birth |
| 67 | 29 | 16+2 | 47,XXY | True positive | 47,XXY | NA | TOP |
| 68 | 28 | 16+4 | 47,XXY | - | NA | Normal | Live birth |
| 69 | 28 | 14+1 | 47,XXY | - | NA | Normal | Live birth |
| 70 | 33 | 16+5 | 47,XXY | True positive | 47,XXY | Normal | TOP |
| 71 | 39 | 17+6 | 47,XXY | - | NA | NA | NA |
| 72 | 36 | 14+5 | 47,XXY | True positive | 47,XXY | Normal | Live birth |
| 73 | 29 | 16+0 | 47,XXY | True positive | 47,XXY | Normal | TOP |
| 74 | 32 | 16+2 | 47,XXY | True positive | 47,XXY | Normal | Live birth |
| 75 | 31 | 16+1 | 47,XXY | - | NA | NA | Live birth |
| 76 | 34 | 15+3 | 47,XXY | - | NA | Normal | Live birth |
| 77 | 38 | 18+0 | 47,XXY | - | NA | NA | Live birth |
| 78 | 32 | 17+0 | 47,XXY | True positive | 47,XXY | NA | TOP |
| 79 | 32 | 17+0 | 47,XXY | True positive | 47,XXY | Normal | TOP |
| 80 | 29 | 13+2 | 47,XXY | True positive | 47,XXY | Normal | TOP |
| 81 | 28 | 15+3 | 47,XXY | True positive | 47,XXY | Normal | TOP |
| 82 | 28 | 16+1 | 47,XXY | - | NA | NA | NA |
| 83 | 34 | 16+3 | 47,XXY | True positive | 47,XXY | Normal | TOP |
| 84 | 37 | 13+4 | 47,XXY | - | NA | Normal | NA |
| 85 | 33 | 16+6 | 47,XXY | True positive | 47,XXY | Normal | NA |
| 86 | 33 | 13+0 | 47,XXY | True positive | 47,XXY | Normal | TOP |
| 87 | 27 | 16+1 | 47,XXX | True positive | 47,XXX | Normal | Live birth |
| 88 | 32 | 12+5 | 47,XXX | True positive | 47,XXX | Normal | TOP |
| 89 | 32 | 22+6 | 47,XXX | - | NA | NA | Live birth |
| 90 | 26 | 19+6 | 47,XXX | True positive | 47,XXX | Normal | Live birth |
| 91 | 38 | 17+5 | 47,XXX | True positive | 47,XXX | Normal | Live birth |
| 92 | 27 | 19+0 | 47,XXX | - | NA | NA | Live birth |
| 93 | 20 | 19+2 | 47,XXX | False positive | 46,XX | Normal | Live birth |
| 94 | 37 | 19+5 | 47,XXX | - | NA | Normal | Live birth |
| 95 | 42 | 12+1 | 47,XXX | True positive | 47,XXX | Normal | TOP |
| 96 | 29 | 21+1 | 47,XXX | - | NA | NA | Live birth |
| 97 | 30 | 17+0 | 47,XXX | False positive | 46,XX | Normal | Live birth |
| 98 | 35 | 16+6 | 47,XXX | - | NA | NA | Live birth |
| 99 | 34 | 19+0 | 47,XXX | False positive | 46,XX | Normal | Live birth |
| 100 | 32 | 18+0 | 47,XXX | True positive | 47,XXX | Normal | Live birth |
| 101 | 31 | 16+0 | 47,XXX | True positive | 47,XXX | Normal | Live birth |
| 102 | 31 | 16+4 | 47,XXX | - | NA | NA | Live birth |
| 103 | 35 | 16+3 | 47,XXX | True positive | 47,XXX | Normal | Live birth |
| 104 | 33 | 18+5 | 47,XXX | - | NA | NA | Live birth |
| 105 | 29 | 16+4 | 47,XXX | True positive | 47,XXX | Normal | Live birth |
| 106 | 28 | 17+3 | 47,XXX | True positive | 47,XXX | Normal | Live birth |
| 107 | 33 | 15+6 | 47,XXX | - | NA | Normal | Live birth |
| 108 | 34 | 15+3 | 47,XXX | True positive | 47,XXX | Normal | Live birth |
| 109 | 27 | 15+6 | 47,XXX | False positive | 46,XX | Normal | Live birth |
| 110 | 32 | 16+4 | 47,XXX | False positive | 46,XX | Normal | Live birth |
| 111 | 32 | 15+2 | 47,XXX | True positive | 47,XXX | Unilateral choroid plexus cyst | Live birth |
| 112 | 28 | 17+4 | 47,XXX | True positive | 47,XXX | Normal | Live birth |
| 113 | 28 | 16+3 | 47,XXX | True positive | 47,XXX | Normal | Live birth |
| 114 | 32 | 12+0 | 47,XXX | - | NA | Normal | Live birth |
| 115 | 46 | 16+0 | 47,XXX | True positive | 47,XXX | Normal | Live birth |
| 116 | 25 | 13+4 | 47,XXX | False positive | 46,XX | Normal | Live birth |
| 117 | 35 | 26+6 | 47,XXX | - | NA | Normal | NA |
| 118 | 34 | 16+2 | 47,XXX | True positive | 47,XXX | VSD 2.4mm | Live birth |
| 119 | 31 | 17+3 | 47,XXX | True positive | 47,XXX | Normal | Live birth |
| 120 | 27 | 16+1 | 47,XXX | False positive | 46,XX | Normal | Live birth |
| 121 | 36 | 17+1 | 47,XXX | - | NA | NA | Live birth |
| 122 | 34 | 16+5 | 47,XXX | True positive | 47,XXX | Normal | TOP |
| 123 | 27 | 14+2 | 47,XXX | True positive | arr[hg19]Xp22.33(168,552_768,147)×1, 600k; Xq25q28(123,590,006_155,233,098)×3, 31.6Mb | Normal | NA |
| 124 | 28 | 16+4 | 45,X | False positive | 46,XX | Normal | Live birth |
| 125 | 32 | 18+3 | 45,X | False positive | 46,XX | FGR | Live birth |
| 126 | 27 | 16+4 | 45,X | True positive | arr[hg19]Xp22.33p22.2(168,551_11,696,586)×1,11.5Mb | VSD 1.8mm | TOP |
| 127 | 32 | 25+0 | 45,X | False positive | 46,XX | Normal | Live birth |
| 128 | 30 | 13+3 | 45,X | False positive | 46,XX | FGR, Polyhydramnios | Live birth |
| 129 | 29 | 17+2 | 45,X | False positive | 46,XX | Normal | Live birth |
| 130 | 36 | 25+3 | 45,X | False positive | 46,XX | Normal | Live birth |
| 131 | 27 | 15+3 | 45,X | - | NA | NA | Live birth |
| 132 | 37 | 16+3 | 45,X | False positive | 46,XX | Normal | Live birth |
| 133 | 28 | 20+3 | 45,X | False positive | 46,XX | Normal | Live birth |
| 134 | 30 | 13+1 | 45,X | False positive | 46,XX | Normal | Live birth |
| 135 | 26 | 17+3 | 45,X | False positive | 46,XX | Normal | Live birth |
| 136 | 29 | 21+4 | 45,X | False positive | 47,XXX | NA | Live birth |
| 137 | 30 | 15+0 | 45,X | False positive | 46,XX | Increased scalp thickness | Live birth |
| 138 | 30 | 19+6 | 45,X | False positive | 46,XX | NA | Live birth |
| 139 | 30 | 13+5 | 45,X | False positive | 46,XY | Bilateral pyelectasis | Live birth |
| 140 | 32 | 12+5 | 45,X | False positive | 46,XX | Normal | Live birth |
| 141 | 27 | 12+4 | 45,X | False positive | 46,XX | Normal | Live birth |
| 142 | 38 | 17+1 | 45,X | False positive | 46,XX | Normal | Live birth |
| 143 | 27 | 18+1 | 45,X | False positive | 46,XX | Normal | Live birth |
| 144 | 31 | 16+6 | 45,X | True positive | 45,X | NA | TOP |
| 145 | 26 | 12+6 | 45,X | - | NA | Normal | Live birth |
| 146 | 30 | 16+1 | 45,X | False positive | 46,XX | Normal | Live birth |
| 147 | 33 | 16+4 | 45,X | - | NA | NA | Live birth |
| 148 | 36 | 16+0 | 45,X | - | NA | Normal | Live birth |
| 149 | 34 | 17+4 | 45,X | False positive | 46,XX | NA | Live birth |
| 150 | 30 | 18+0 | 45,X | False positive | 46,XX | Normal | Live birth |
| 151 | 33 | 19+1 | 45,X | False positive | 46,XX | FGR | Live birth |
| 152 | 24 | 17+6 | 45,X | False positive | 46,XX | NA | Live birth |
| 153 | 39 | 16+0 | 45,X | False positive | 46,XX | Normal | Live birth |
| 154 | 32 | 16+2 | 45,X | False positive | 46,XX | Normal | Live birth |
| 155 | 32 | 18+6 | 45,X | False positive | 46,XX | Normal | Live birth |
| 156 | 30 | 17+1 | 45,X | False positive | 46,XX | Single umbilical artery | Live birth |
| 157 | 38 | 16+5 | 45,X | True positive | 45,X[6]/47,XXX[44] | NA | Live birth |
| 158 | 34 | 18+2 | 45,X | True positive | 45,X[15]/46,XX[45] | Shortened long bones | TOP |
| 159 | 27 | 12+2 | 45,X | - | NA | Normal | Live birth |
| 160 | 26 | 16+1 | 45,X | True positive | 45,X[29]/46,X,del(X)(p22.31)[21] | Normal | TOP |
| 161 | 27 | 17+3 | 45,X | False positive | 46,XX | Normal | Live birth |
| 162 | 33 | 16+4 | 45,X | False positive | 46,XX | Mild pulmonary valve regurgitation | Live birth |
| 163 | 33 | 13+2 | 45,X | False positive | 46,XX | Normal | Live birth |
| 164 | 28 | 16+4 | 45,X | False positive | 46,XX | Normal | Live birth |
| 165 | 30 | 18+1 | 45,X | False positive | 46,XX | Normal | Live birth |
| 166 | 27 | 17+3 | 45,X | True positive | arr[hg19](1-22)×2,(X)×1~2 | NA | TOP |
| 167 | 26 | 17+3 | 45,X | False positive | 46,XX | Normal | Live birth |
| 168 | 30 | 15+6 | 45,X | False positive | 46,XX | Normal | Live birth |
| 169 | 33 | 19+6 | 45,X | True positive | arr[hg19]Xq13.3q23(74,261,328_113,950,436)×1,39.7Mb | Normal | TOP |
| 170 | 29 | 13+2 | 45,X | False positive | 46,XX | Normal | Live birth |
| 171 | 28 | 15+1 | 45,X | False positive | 46,XX | Normal | Live birth |
| 172 | 30 | 12+4 | 45,X | - | NA | Normal | Live birth |
| 173 | 28 | 12+5 | 45,X | False positive | 46,XX | NA | Live birth |
| 174 | 28 | 16+5 | 45,X | False positive | 46,XX | Normal | Live birth |
| 175 | 28 | 19+3 | 45,X | False positive | 46,XX | Normal | Live birth |
| 176 | 29 | 15+1 | 45,X | True positive | 45,X[6]/46,XX[44] | Normal | TOP |
| 177 | 28 | 18+1 | 45,X | True positive | 45,X[40]/46,XY[10] | Normal | TOP |
| 178 | 33 | 12+6 | 45,X | False positive | 46,XX | NA | Live birth |
| 179 | 30 | 15+5 | 45,X | False positive | 46,XX | NA | Live birth |
| 180 | 41 | 16+6 | 45,X | - | NA | Normal | Live birth |
| 181 | 25 | 13+2 | 45,X | False positive | 46,XX | FGR | Live birth |
| 182 | 33 | 17+1 | 45,X | False positive | 47,XXX | Normal | TOP |
| 183 | 26 | 14+2 | 45,X | False positive | 46,XX | NA | Live birth |
| 184 | 27 | 14+4 | 45,X | - | NA | Normal | TOP |
| 185 | 31 | 12+2 | 45,X | False positive | 46,XX | Normal | Live birth |
| 186 | 28 | 14+3 | 45,X | True positive | 45,X | Normal | TOP |
| 187 | 31 | 13+4 | 45,X | False positive | 46,XX | Normal | Live birth |
| 188 | 28 | 23+6 | 45,X | False positive | 46,XX | Normal | Live birth |
| 189 | 35 | 17+5 | 45,X | - | NA | NA | Live birth |
| 190 | 34 | 19+4 | 45,X | True positive | 45,X | Normal | TOP |
| 191 | 30 | 16+0 | 45,X | False positive | 46,XX | Polyhydramnios | Live birth |
| 192 | 28 | 16+1 | 45,X | False positive | 46,XX | NA | Live birth |
| 193 | 31 | 17+1 | 45,X | True positive | 45,X[11]/46,XX[39] | NA | TOP |
| 194 | 31 | 16+2 | 45,X | False positive | 46,XX | Normal | NA |
| 195 | 29 | 17+0 | 45,X | - | NA | Normal | NA |
| 196 | 28 | 17+0 | 45,X | True positive | 45,X[9]/46,XX[41] | Normal | Live birth |
| 197 | 31 | 16+5 | 45,X | False positive | 46,XX | Normal | Live birth |
| 198 | 30 | 23+4 | 45,X | False positive | 46,XX | Normal | Live birth |
| 199 | 38 | 13+0 | 45,X | - | NA | Normal | Live birth |
| 200 | 33 | 15+6 | 45,X | - | NA | NA | NA |
| 201 | 34 | 16+4 | 45,X | False positive | 46,XX | Battledore placenta | Live birth |
| 202 | 39 | 16+4 | 45,X | False positive | 46,XX | Normal | Live birth |
| 203 | 30 | 17+0 | 45,X | False positive | 46,XX | Normal | Live birth |
| 204 | 30 | 15+4 | 45,X | False positive | 46,XX | Normal | Live birth |
| 205 | 27 | 15+3 | 45,X | False positive | 46,XX | Normal | Live birth |
| 206 | 25 | 17+5 | 45,X | False positive | 46,XX | NA | Live birth |
| 207 | 28 | 16+3 | 45,X | False positive | 46,XX | NA | NA |
| 208 | 31 | 16+4 | 45,X | False positive | 46,XX | NA | Live birth |
| 209 | 31 | 16+0 | 45,X | True positive | arr[hg19]Xp22.33p22.31(168,552_6,771,420)×1,6.6Mb | Shortened long bones | TOP |
| 210 | 32 | 14+2 | 45,X | False positive | 46,XX | Normal | Live birth |
| 211 | 30 | 19+0 | 45,X | True positive | 45,X[15]/46,XX[45] | NA | TOP |
| 212 | 34 | 18+2 | 45,X | False positive | 46,XX | Normal | Live birth |
| 213 | 32 | 19+1 | 45,X | False positive | 46,XX | Normal | Live birth |
| 214 | 40 | 16+2 | 45,X | True positive | 45,X[21]/46,XX[39] | NT 3.1mm | Live birth |
| 215 | 34 | 15+5 | 45,X | True positive | 45,X[12]/46,XX[48] | Normal | Live birth |
| 216 | 28 | 23+1 | 45,X | - | NA | Normal | Live birth |
| 217 | 29 | 12+4 | 45,X | False positive | 46,XX | Normal | Live birth |
| 218 | 28 | 17+3 | 45,X | False positive | 46,XX | Normal | Live birth |
| 219 | 26 | 16+5 | 45,X | False positive | 46,X,idic(Y)(q11.22) | Normal | TOP |
| 220 | 29 | 17+1 | 45,X | - | NA | Normal | Live birth |
| 221 | 20 | 19+1 | 45,X | False positive | 46,XX | NA | NA |
| 222 | 33 | 16+5 | 45,X | False positive | 46,XX | Normal | Live birth |
| 223 | 30 | 19+0 | 45,X | False positive | 46,XX | Normal | NA |
| 224 | 29 | 21+0 | 45,X | False positive | 46,XX | Normal | NA |
| 225 | 32 | 17+1 | 45,X-M | False positive | 46,XY | NA | Live birth |
| 226 | 28 | 15+4 | 45,X-M | False positive | 46,XX | Normal | Live birth |
| 227 | 31 | 16+1 | 45,X-M | False positive | 46,XX | Normal | Live birth |
| 228 | 25 | 17+4 | 45,X-M | False positive | 46,XY | Normal | Live birth |
| 229 | 27 | 16+1 | 45,X-M | False positive | 46,XX | Normal | Live birth |
| 230 | 28 | 16+4 | 45,X-M | False positive | 46,XY | Normal | Live birth |
| 231 | 30 | 16+6 | 45,X-M | False positive | 46,XY | Normal | Live birth |
| 232 | 28 | 17+1 | 45,X-M | - | NA | Normal | Live birth |
| 233 | 28 | 13+2 | 45,X-M | False positive | 46,XY | Normal | Live birth |
| 234 | 36 | 18+4 | 45,X-M | - | NA | Normal | Live birth |
| 235 | 30 | 19+2 | 45,X-M | - | NA | Normal | Live birth |
| 236 | 30 | 17+3 | 45,X-M | False positive | 46,XX | NA | Live birth |
| 237 | 33 | 16+4 | 45,X-M | - | NA | Normal | Live birth |
| 238 | 27 | 13+1 | 45,X-M | - | NA | NA | NA |
| 239 | 32 | 17+0 | 45,X-M | - | NA | Normal | Live birth |
| 240 | 32 | 19+4 | 47,XXX-M | False positive | arr[hg19](1-22,X)×2 | NA | Live birth |
| 241 | 25 | 14+2 | 47,XXX-M | False positive | 46,XY | Normal | Live birth |
| 242 | 34 | 20+4 | 47,XXX-M | - | NA | Normal | Live birth |
| 243 | 29 | 20+6 | 47,XXX-M | - | NA | Normal | Live birth |
| 244 | 31 | 24+5 | 47,XXX-M | - | NA | NA | NA |
| 245 | 24 | 12+5 | 47,XXX-M | False positive | 46,XX | Normal | Live birth |
| 246 | 25 | 18+0 | 47,XXX-M | False positive | 46,XX | Normal | Live birth |
| 247 | 24 | 17+5 | 47,XXX-M | - | NA | Normal | Live birth |
| 248 | 20 | 18+3 | 47,XXX-M | - | NA | NA | Live birth |
| 249 | 25 | 16+0 | 47,XXX-M | False positive | 46,XX | Normal | Live birth |
| 250 | 37 | 15+6 | 47,XXX-M | - | NA | NA | Live birth |
| 251 | 35 | 17+2 | 47,XXX-M | False positive | 46,XX | Normal | Live birth |
| 252 | 33 | 17+1 | 47,XXX-M | False positive | 46,XX | Normal | Live birth |

FGR, fetal growth restriction; SCAs, sex chromosome aneuploidies; VSD, ventricular septal defect; TOP, termination of pregnancy; M, maternal.
